# Highly Pathogenic Porcine Reproductive and Respiratory Syndrome, China

**DOI:** 10.3201/eid1309.070399

**Published:** 2007-09

**Authors:** Guang-Zhi Tong, Yan-Jun Zhou, Xiao-Fang Hao, Zhi-Jun Tian, Tong-Qing An, Hua-Ji Qiu

**Affiliations:** * Harbin Veterinary Research Institute–Chinese Academy of Agricultural Sciences, Harbin, People’s Republic of China

**Keywords:** Porcine, reproductive, respiratory, China, letter

**To the Editor:** Since April 2006, a highly pathogenic disease caused by unknown agents and characterized by high fever and a high proportion of deaths in pigs of all ages, emerged in some swine farms in Jiangxi Province, People’s Republic of China. The morbidity rate was 50%–100% and mortality rate was 20%–100%. In the next several months, the disease spread rapidly to most provinces of China. In almost all affected swine herds, the following clinical signs were observed: high and continuous fever, anorexia, red discolorations in the bodies, and blue ears; in the late phase of the disease, diarrhea and other clinical signs might be seen due to the secondary infections. Clinical samples (from lungs, kidneys, liver, and lymph nodes) were collected from animals in different provinces and sent for laboratory diagnosis. DNA and RNA were extracted from the tissue homogenate and PCR or reverse transcription–PCR (RT-PCR) was conducted to detect porcine reproductive and respiratory syndrome virus (PRRSV), classic swine fever virus, porcine circovirus, and pseudorabies virus, respectively (*1*). In clinical samples, only PRRSV was found to be the dominant virus (48 of 50 samples were PRRSV positive). PRRSVs were then isolated successfully on MARC-145 cells with an obvious cytopathologic effect, characterized by cell congregation, contraction, and brushing off at passage 2; immunofluorescence assay using PRRSV NP-, M- and GP5-specific monoclonal antibodies confirmed that the isolated viruses were PRRSV (*2*,*3*). Full-length genomic sequencing of 1 of the isolates (HuN4 strain) showed extensive amino acid (aa) mutations in GP5 protein and 2 deletions in Nsp2, 1 aa deletion at 482, and 29 aa deletions at 533–561, compared with the previous Chinese isolates CH-1a and BJ-4.

The newly isolated PRRSV was used to examine the pathogenicity in 60-day-old PRRSV-free piglets, under closed and biosafety (P2) conditions. Each of the piglets (N = 5) received intranasally 10^5.0^ 50% tissue culture infecting dose of the isolated virus propagated in MARC-145 cells (*4*,*5*). The animals were kept in separate rooms throughout the experiment. Clinical observations of respiratory signs, behavior, rectal temperature, and coughing were recorded daily. Blood samples were collected every 2 days and tested for PRRSV-specific antibodies by ELISA (*6*,^7^). Tissue samples (from heart, lungs, kidneys, spleen, and lymph nodes) from all animals that died during the experiment were collected and detected by histopathologic examination (*8*) and virus isolation. Results showed that the clinical manifestations of all pigs were similar to those that appeared in the field investigation (including high and continuous fever, anorexia, red discolorations in the bodies, and blue ears). The specific antibodies to PRRSV were detected at 8 days postinfection, and the high antibody level lasted until the animal’s death, and all infected pigs died at either 7, 8, 12, 16, or 21 days postinoculation, respectively. Furthermore, viruses reisolated from the dead pigs showed an identical homology with the inoculated PRRSV in genes coding for GP5 and partial Nsp2 (2,535–3,307 nt). The results showed that the emerging PRRSV, characterized by deletions in Nsp2, is highly pathogenic to pigs.

To investigate whether the emerging PRRSV was the causative agent of the pandemic diseases on swine farms, an extensive virus survey was conducted. More than 48 samples collected from different swine farms in12 provinces were found to be PRRSV positive by RT-PCR, based on open reading frame (ORF) 5 and Nsp2 (Figure). Sequence analysis of ORF5 and partial Nsp2 showed that these PRRSVs are highly homologous to each other (98.5%–100% for GP5; 98.2%–100% for Nsp2) and share the same deletions at the same positions of Nsp2 gene with HuN4 strain. Sequence comparison of ORF5 indicated that the HuN4 strain shares 93%, 86%, and 88% nucleotide identities with CH-1a (Chinese isolate), BJ-4 (Chinese isolate), and VR2332 (American isolate), respectively. All the newly isolated PRRSVs belong to the North American type.

**Figure:**
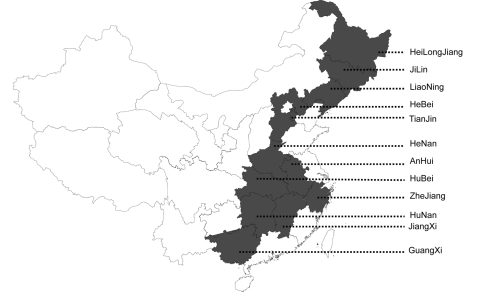
Geographic distribution of porcine reproductive and respiratory syndrome viruses (PRRSVs) examined in the study. Shaded areas indicate the provinces where the PRRSVs characterized by deletions in Nsp2 were detected.

Although the cause of the emerging pandemic disease of pigs with a high proportion of deaths in 2006 is unknown, we found high correlation between PRRSV isolation rate and the diseased pigs. The regression test in its natural animal showed that the newly isolated PRRSV was much more virulent than earlier PRRSV isolates. Also, sequence analysis demonstrated a substantial diversity from the PRRSVs isolated during 1996–2005. Further study is needed to answer the question: What role did the newly isolated PRRSV play in the 2006 outbreaks on many of the swine farms in China?
